# A novel ECG-biomarker for cardiac arrest during hypothermia

**DOI:** 10.1186/s13049-020-00721-0

**Published:** 2020-04-10

**Authors:** Erik Sveberg Dietrichs, Torkjel Tveita, Rachel Myles, Godfrey Smith

**Affiliations:** 1 Experimental and Clinical Pharmacology Research Group, Department of Medical Biology, UiT, The Arctic University of Norway, 9037 Tromsø, Norway; 2 Department of Clinical Pharmacology, Division of Diagnostic Services, University Hospital of North Norway, 9038 Tromsø, Norway; 3 Anesthesia and Critical Care Research Group, Department of Clinical Medicine, UiT, The Arctic University of Norway, 9037 Tromsø, Norway; 4 Division of Surgical Medicine and Intensive Care, University Hospital of North Norway, 9038 Tromsø, Norway; 5 Institute of Cardiovascular & Medical Sciences, University of Glasgow, Glasgow, UK

**Keywords:** Hypothermia, Electrophysiology, Ventricular arrhythmias, Therapeutic hypothermia, Cardiac arrest

## Abstract

**Background:**

Treatment of arrhythmias evoked by accidental or therapeutic hypothermia and rewarming remains challenging. We aim to find an ECG-biomarker that can predict ventricular arrhythmias at temperatures occurring in therapeutic and accidental hypothermia.

**Main body:**

Evaluation of ECG-data from accidental and therapeutic hypothermia patients and experimental data on ECG and ventricular fibrillation (VF) threshold in hypothermic New Zealand White Rabbits. VF threshold was measured in rabbit hearts cooled to moderate (31 °C) and severe (17 °C) hypothermia. QRS-interval divided by corrected QT-interval (QTc) was calculated at same temperatures. Clinical QRS/QTc data were obtained after a systematic literature review. Rabbit QRS/QTc values correlated with risk for VF (correlation coefficient: 0.97). Human QRS/QTc values from hypothermic patients, showed similar correlation with risk for ventricular fibrillation in the experimental data (correlation coefficient: 1.00).

**Conclusions:**

These calculations indicate that QRS/QTc has potential as novel biomarker for predicting risk of hypothermia-induced cardiac arrest. Our findings apply both to victims of accidental hypothermia and to patients undergoing therapeutic hypothermia during surgery or after e.g. cardiac arrest.

## Background

Accidental hypothermia is a severe condition with high mortality rate, ranging between 25 and 40% in most studies [1]. In young patients succumbing to accidents at sea or harsh weather conditions, many life-years are lost. It is however possible to survive extreme exposure if correct treatment is provided. Hypothermia lowers metabolism and is neuroprotective, allowing survival after accidental cooling down to a core temperature of 13.7 °C [2]. Hypothermic patients are however at grave risk of developing refractory ventricular fibrillation (VF) and cardiac arrest with little chance of successful defibrillation during evacuation and transport to hospital [3]. Such witnessed hypothermic cardiac arrest is termed “rescue collapse” and Frei et al. found an associated mortality rate of 27% [4]. The pathophysiology has been largely unknown and it is therefore challenging to predict arrhythmias and rescue collapse in hypothermic patients, which is related to movement during extrication, mobilisation or transfer [4].

## Main text

In a recent study [5], we found that cooling of rabbit hearts to mild-moderate hypothermia (31 °C) alters ventricular repolarisation while transmural conduction remains relatively unchanged. Rabbits were chosen do to the close resemblance to human cardiac electrophysiology [6]. When provoking arrhythmias by electrical stimulation in the rabbit model, we found that this temperature-dependent combination of effects increased risk for VF (decreased VF threshold) in moderate hypothermia and was pro-arrhythmic. Exposure to severe hypothermia (17 °C), conversely, decreased risk for VF as conduction and repolarisation was equally affected. These changes were reflected in QRS and QT-intervals on the ECG. Correcting the QT interval for heart rate reinforced the association between ECG-findings and pro-arrhythmic activity during hypothermia. We found that relative values of QRS-intervals to the corrected QT-interval, correlates with increased risk for ventricular arrhythmia in moderate hypothermia.

**Fig. 1:**
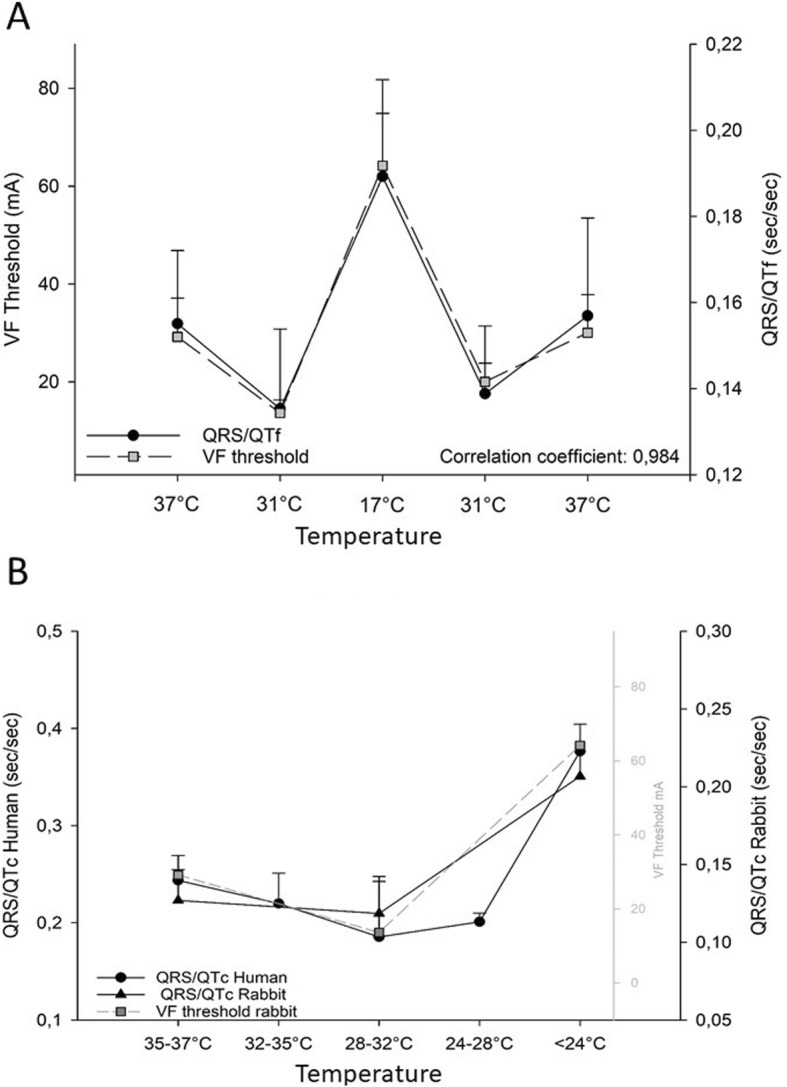
**a** We have found four potential biomarkers that correlate with risk for cardiac arrest in hypothermic rabbit hearts, calculated from QRS interval relative to Bazett’s (QTc) and Fredericia’s (QTf) correction of QT-interval. QRS/QTf is the most promising experimental biomarker. **b** QRS/QTc data from the included studies plotted against QRS/QTc and VF threshold values from rabbit. Human data showed high correlation (comparing 37 °C, 28–32 °C and < 24 °C) with both rabbit QRS/QTc (correlation coefficient: 0.97) and rabbit VF threshold (correlation coefficient: 1.00). Human values are given as mean (weighted for number of patients in each study) ± SD between study means weighted for number of patients in each study

Based on these results, we found two biomarkers that correlate highly (correlation coefficient 0.97–0.98) with risk for cardiac arrest in hypothermic hearts, using Bazett’s (QTc) or Fredericia’s (QTf) correction of QT-interval. We find that QRS/QTc emerge as the most available clinical biomarker. It has a comparable correlation (0.97) with VF threshold and is available for calculation from previously published clinical data (Fig. 1).

To assess the clinical value of QRS/QTc in hypothermic patients, we extracted data from a recently published systematic review article of hypothermia and cardiac electrophysiology [1]. In a total of 8 studies on accidental and therapeutic hypothermia, QRS- and QTc-intervals were listed from patients that could be categorized (Table 1) into normothermia (35–37 °C) and varying degrees of hypothermia: 32–35 °C (mean 33.1 °C), 28–32 °C (mean 30.6 °C), 24–28 °C (mean 26.7 °C), and < 24 °C (mean 23.3 °C). As expected, at the lowest temperatures we found data from few patients, which must be taken account for when assessing QRS/QTc values from < 24 °C and 24–28 °C.

**Table 1:** In a total of 8 studies [1] on accidental and therapeutic hypothermia, QRS- and QTc-intervals were published from patients that could be categorized into normothermia and varying degrees of hypothermia. Human values are given as mean (weighted for number of patients)

Temperature	QRS mean (sec)	QTc mean (sec)	QRS/QTc	QTc/QRS	Patients ***n***=	Included studies ***n***=
**35–37 °C**	0,114	0,468	0,244	4,11	371	5
**32–35 °C**	0,113	0,515	0,220	4,56	282	5
**28–32 °C**	0,097	0,520	0,187	5,36	156	4
**24–28 °C**	0,114	0,565	0,201	4,96	20	2
**< 24 °C**	0,160	0,425	0,389	2,66	2	1

In Fig. 1 we present QRS/QTc data from the included clinical studies plotted against QRS/QTc and VF threshold values from rabbit. Human data showed high correlation with both rabbit QRS/QTc (correlation coefficient: 0.97) and rabbit VF threshold (correlation coefficient: 1.00). This implies that QRS/QTc could predict risk for VF at different temperatures in hypothermic patients. As rescue collapse contributes to the high mortality rate in accidental hypothermia [4], prediction of VF risk could be of high clinical value. This would be relevant also in the in-hospital setting, where QRS/QTc could have a role in assessing safety of therapeutic hypothermia treatment. The inverse calculation; QTc/QRS could be an even more easily accessible clinical marker, relating higher values with higher risk for VF during hypothermia.

## Conclusion

We believe that these calculations, as we have applied on preclinical and clinical data, have potential as novel biomarkers for predicting risk of hypothermia-induced cardiac arrest. QTc/QRS or QRS/QTc could easily be tested in the clinic, and have potential to be implemented in guidelines to predict rescue collapse and ease further clinical research into pharmacological prevention of this condition. Our findings apply both to victims of accidental hypothermia and to patients undergoing therapeutic hypothermia during surgery or after e.g. cardiac arrest, where a biomarker used for risk assessment would be of high value.

## Data Availability

The datasets during and/or analysed during the current study available from the corresponding author on reasonable request.
